# Comparing the effects of two different contact lenses on corneal re-epithelialization after corneal collagen cross-linking

**DOI:** 10.12669/pjms.333.12241

**Published:** 2017

**Authors:** Yusuf Kocluk, Savas Cetinkaya, Emine Alyamac Sukgen, Murat Günay, Alper Mete

**Affiliations:** 1 Yusuf Kocluk, MD. Adana Numune Training and Research Hospital, Ophthalmology Clinic, Adana, Turkey; 2 Savas Cetinkaya, MD. Adana Numune Training and Research Hospital, Ophthalmology Clinic, Adana, Turkey; 3 Emine Alyamac Sukgen, MD Adana Numune Training and Research Hospital, Ophthalmology Clinic, Adana, Turkey; 4 Murat Günay, MD. Ophthalmology Clinic, Zeynep Kamil Gynecology and Pediatrics Education and Research Hospital, Istanbul, Turkey; 5 Alper Mete, MD. Gaziantep University, School of Medicine, Department of Ophthalmology, Gaziantep, Turkey

**Keywords:** Keratoconus, Corneal collagen cross-linking, Bandage contact lens, Corneal re-epithelialization, Allergic conjunctivitis

## Abstract

**Objective:**

To investigate whether keratoconus (KC) patients who applied the corneal collagen cross-linking (CXL) and two different contact lens (CL) showed any differences in complaints and findings following the CXL.

**Methods:**

This prospective, comparative, double-blind clinical study involved 60 eyes of 60 patients (38 female and 22 male). At the end of the CXL procedure, CL (Balafilcon A) was inserted in 29 patients (Group-1) while CL with different material content (Hioxifilcon A) designed for therapeutic/bandage purposes were inserted in 31 patients (Group-2).

**Results:**

On the 1^st^ and 3^th^ day after the CXL, there were no statistically significant differences between the groups in terms of the postoperative symptoms. On the 3^th^ day after the CXL, all cases of both two groups were found to complete the corneal re-epithelialization. There was more PE ratio in the patients who had allergic conjunctivitis.

**Conclusions:**

With the use of Balafilcon A and Hioxifilcon A lens materials, KC patients who underwent the CXL were found to have similar symptoms and clinical findings after the CXL. However, epithelial staining and PE were observed more in KC cases accompanied by allergic conjunctivitis.

## INTRODUCTION

Progressive keratoconus (KC) requires making corneal collagen cross-linking (CXL) through which the mechanical strength and biochemical stability of the cornea is enhanced. Until now, it has been the only treatment which addresses pathophysiology of KC.^1^-^4^ Final phase of the CXL treatment includes the application of a bandage contact lens (B-CL) until the epithelium is completely healed. It was combined with the application of topical corticosteroids, antibiotic, and non-steroidal anti-inflammatory agents.^5^

Remarkable adverse effects are noted in the eyes with removed epithelium of cornea. Some of these effects include prolonged visual recovery because of the process of epithelial wound healing and severe pain, usually starting on the day of surgery and continuing until corneal re-epithelialization.^6^ Silicone hydrogel contact lenses (SH-CL) are utilised as bandage purpose with a view to decreasing the average time for epithelial healing, improving visual acuity, and controlling surface generated pain followed by some corneal surgery.^7^,^8^ Risk factor for postoperative infections can be decreased, earlier visual rehabilitation can be facilitated, and patient comfort can be increased by providing faster re-epithelialization.^8^

Various soft CL materials for bandage use have been recommended after corneal refractive surgery.^9^ Studies that compare re-epithelialization and postoperative complaints according to the use of different materials following a reactive surgery can be found in the related literature.^6^-^9^ On the other hand, no studies seem to have investigated whether re-epithelialization and subjective complaints changed after the CXL, with CL made of different materials or with B-CL produced specifically for therapeutic/bandage purposes.

This study investigated whether KC patients who used the CXL and two different CLs (Balafilcon A and Hioxifilcon A) showed any differences in complaints following the CXL and the time of corneal re-epithelialisation.

##  METHOD

This prospective, comparative, double-blind clinical study involved 60 eyes of 60 patients (38 female and 22 male). Average age of the patients was found 18.1±4.5 years (range: 12–28 years). Unilateral CXL was applied to all patients for progressive KC in an ophthalmology clinic of a tertiary care center between June 2015 and February 2016. All patients’ gave written informed consent, approval of hospital ethics committee was received, and the study followed the principles of the Declaration of Helsinki.

At the end of the CXL procedure, SH-CL (Balafilcon A material, Bausch & Lomb Pure Vision, Rochester, NY) was inserted in 29 patients (group 1) while B-CL with different material content (Polymer based on GMMA [Hioxifilcon A] material, Interojo Inc, Korea) particularly designed for therapeutic/bandage purposes were inserted in 31 patients (group 2). Inserting the CL was done in a consecutive sequence. CL details are shown in Table-I.

**Table-I:** Details of contact lenses.

*Parameters*	*Silicone Hydrogel CL*	*Bandage CL (For eye surgery)*
Base curve	8.6	Bi-curve lens (Center part: 9.0, peripheral part:8.6)
Diameter	14.0	14.2
Power	Plano	Plano
Water content	36%	55%
Material	Balafilcon A	Polymer based on GMMA(Hioxifilcon A)
DK	91	25
Replacement	Monthly	Monthly
Manufacturer	Bausch & Lomb	Interojo

CL: Contact Lens

Before the CXL treatment, all the patients underwent full ophthalmic examination and corneal topography. Topographic analysis was obtained through Pentacam (Oculus Optikgerate, Wetzlar, Germany). Biomicroscopic examinations were done. Patients were followed up on the 1^st^, 3^th^, and 10^th^ day after the CXL treatment. Absence of postoperative infection was also monitored.

CL was removed on the 3^th^ examination day. On the postoperative 3^th^ and 10^th^ days, corneal staining was performed with fluorescein; and whether corneal re-epithelialization was completed or not was monitored. Existence of punctate epitheliopathy (PE) was checked and scored. PE was scored between grade 0 and 3; meaning grade 0: no epitheliopathy, Grade-1: under 25% of corneal surface, grade 2: between 25% and 50% of corneal surface, and Grade-3: over 50% of corneal surface. The examinations also included checking the existence of any stromal infiltrate as a side effect of the CXL.

Postoperative complaints assessed in the examinations included ocular pain, eyelid edema, conjunctival hyperemia, and ocular irritation with burning. Scoring of the parameters were done as 0:no, 1:slight, 2: moderate, 3: severe. The same surgeon (S.C) performed all the CXL treatments, and the same cornea specialist (Y.K) did all the examinations before and after the CXL. During the examinations, he did not know which CL was inserted at operation.

Progression of KC in the preoperative months (change in K-max value > 1dpt, thinning of the cornea by > 30μm, increase of topographical astigmatism by > 1dpt) and KC in age under 20 were the inclusion criteria for the CXL treatment in the present study.

Exclusion criteria were corneal thickness < 370 μm at thinnest location, corneal epithelial healing disorders, previous herpes keratitis, corneal melting disorders, pregnancy, continuous eye rubbing habits (especially when associated with down syndrome, CL wearing, floppy eyelid syndrome, and nervous habitual eye rubbing) and corneal scarring.

The patients who had active and symptomatic allergic conjunctivitis findings before the CXL were given treatment and included in the study after the findings were ameliorated with appropriate medication.

### Accelerated CXL procedure

Proparacaine 0.5% eye drops (Alcaine, Alcon Laboratories, Inc.) was administered for topical anaesthesia. Then using a blunt blade, the epithelium was removed under sterile conditions in a central area of 8.0 mm diameter. Every three minutes for 30 minutes, 0.1% riboflavin solution (Merribo iso-osmalar, Meran Medical, Istanbul, Turkey) was applied to the cornea as a photosensitizer. For thin cornea (370-420 μm at thinnest location), Hipo-osmalar 0.1% riboflavin (Merribo hipo-osmalar, Meran Medical, Istanbul, Turkey) was preferred. Following these, UVA light (Apollon Cross-linking System, İstanbul, Turkey) at a wavelength of 370 nm and an irradiance of 9 mW/cm^2^ was used for 10 minutes for irradiating the central cornea. The cornea was applied riboflavin solution every two minutes for 10 minutes during UVA irradiation. A working distance of 5 cm from the corneal surface was set for the device.

At the final phase of the procedure, SH-CL (Balafilcon A material) was inserted on cornea in Group-1 (29 patients) and another type of CL (Polymer based on GMMA [Hioxifilcon A] material) was inserted on cornea in Group-2 (31 patients).

Both groups were administered the same postoperative medication regimen; they were given Moxifloxacin 0.5% eye drops (Vigamox, Alcon) four times a day for two week, topical loteprednol (Lotemax ophthalmic suspension, Bausch & Lomb) 4 times a day in tapering doses for 4 weeks, and topical artificial tear supplements four times a day for one month. On the 3^th^ postoperative day, the CL was removed.

### Statistical Analysis

The data obtained from the study were analyzed using SPSS version 16 software. Where appropriate, chi-square, fisher’s exact test and independent sample t-tests were used. Significance was taken as p value < 0.05.

## RESULTS

The mean age of the participants was found 18.1±4.7 years and 18.2±4.5 years for Group- 1 and Group-II respectively (p= 0.941). The female/male ratio was 19/10 in Group-I and 19/12 in group 2 (p=0.734). No significant differences were detected between the groups in terms of the laterality (p=0.611). 8 (27.6%) of the cases in Group-I and 5 (16.1%) of the cases in Group-II were found to have allergic conjunctivitis non symptomatic under anti allergic treatment before the CXL. The groups did not have any significant differences in terms of the allergies (p=0.282).

According to Amsler-Krumeich classification of KC on Pentacam topography system, preoperative stage of KC did not indicate any significant differences between the groups (p=0.858). 12 (41.4%) cases in Group-I were stage 2, and 16 (55.2%) cases were stage 3; only one case (3.4%) was stage 4. As for Group-II, 13 (41.9%) cases were stage 2, 16 (51.6%) cases were stage 3, and 2 (6.5%) cases was stage 4.

On the 1^st^ and 3^th^ day after the CXL, there were no statistically significant differences between the groups in terms of the postoperative symptoms. Table-II demonstrates the evaluation and scores of the parameters with p values.

**Table-II:** Comparison of signs and symptoms in groups at first and third day after corneal collagen cross-linking.

*Finding and symptoms*	*Group 1(Balafilcon A)*	*Group 2 (Hioxifilcon A)*	*P value*
***First day after CXL***	*no/slight/moderate/severe (%)*		

Ocular pain	55.2%/41.4%/3.4%/0%	48.4%/41.9%/6.5%/3.2%	0.720
Eyelid edema	37.9%/44.8%/17.2%/0%	35.5%/61.3%/3.2%/0%	0.155
Conjunctival hyperemia	17.2%/31.0%/48.3%/3.4%	12.9%/61.3%/25.8%/0%	0.100
İrritation and burning	20.7%/79.3%/0%/0%	32.3%/67.7%/0%/0%	0.311
***Third day after CXL***			
Ocular pain	100%/0%/0%/0%	93.5%/6.5%/0%/0%	0.492
Eyelid edema	100%/0%/0%/0%	96.8%/3.2%/0%/0%	0.329
Conjunctival hyperemia	100%/0%/0%/0%	93.5%/6.5%/0%/0%	0.164
İrritation and burning	86.2%/13.8%/0%/0%	96.8%/3.2%/0%/0%	0.188

CXL: Corneal collagen cross-linking

On the 3^th^ day after the CXL, cornea was stained with fluorescein after the removal of CL. All cases of both two groups were found to complete the corneal re-epithelialization. However, some patients in the groups were observed to have PE. On the 3^th^ day following the CXL, 7 (24.1%) cases had Grade-1 PE and 6 (20.7%) cases had grade 3 PE in Group-I. As to group 2, 9 (29.0%) cases had Grade-1 PE, 3 (9.7%) cases had grade 2 PE and 1 (3.2%) case had grade 3 PE. The groups were not statistically different in terms of the PE (p=0.076).

The PE finding was compared in term of the presence or absence of allergic conjunctivitis. 13 (21.7%) of 60 patients had allergic conjunctivitis. There was more PE ratio in the patients who had allergic conjunctivitis. Distribution of PE in patients was presented in table-III. The difference was statistically significance (p<0.001).

**Table-III:** Comparison of the punctate epitheliopathy according the presence or absence of allergic conjunctivitis at third day after corneal collagen cross-linking.

	*Presence of AC*	*Absence of AC*	*P value*
Grading of PE			<0.001
No PE	2 (15.4%)	32 (68.1%)	
Grade 1 PE	3 (23.1%)	13 (27.7%)	
Grade 2 PE	1 (7.7%)	2 (4.3%)	
Grade 3 PE	7 (53.8%)	0 (0%)	

AC: allergic conjunctivitis

On the 10^th^ day after the CXL, no patients in both groups were found to have PE. On the other hand, one patient with allergic conjunctivitis was diagnosed with sterile stromal infiltrate in Group-II. The difference was not statistically significant between the groups (p=0.517). None of the patients in both groups were found to have infectious keratitis during the re-epithelialisation after the CXL.

## DISCUSSION

The central 8–9 mm of the epithelium is removed in the standard CXL protocol first decribed by Wollensak and colleagues.^10^ This beneficial procedure, which was proven to cease progression in KC patients, continues to cause infectious risks in the period when corneal epithelium is patent. Recently, Boxer Wachler et al. suggested a modification of the technique by keeping the epithelium intact, named epithelium-on or transepithelial CXL.^11^ In the standard CXL, materials that may decrease the speed of corneal re-epithelialisation and increase inflammation should be avoided.

Re-epithelialisation of the cornea is frequently assisted in practice by the use of soft CL as B-CL. The lens can be either a conventional hydrogel or a silicone hydrogel.^12^,^13^ Faster and more effective re-epithelialisation can occur if more oxygen is available.^14^ The cornea will obtain more oxygen with higher Dk of a contact lens. Although the issues related to corneal hypoxia were resolved with the introduction of silicone hydrogel lenses, the risk of microbial keratitis was not reduced.^15^ Instead, increased incidence and risk of inflammatory events have been associated with silicone hydrogel lenses.^16^ In the present study, we investigated the effect of two different CLs having different Dk, water content and materials and inserted for three days following the CXL on corneal re-epithelisation and postoperative symptoms. Similar patient complaints were identified on the first postoperative day in the study. Better results were observed in the comparison of 3^th^ day examination in the patient group in which SH-CL was used clinically; but these differences were not found to be statistically significant.

The risks associated with hypoxia, edema, microbial keratitis and neovascularisation will be decreased with the increase in oxygen transmissibility. The rate of microbial keratitis in extended wear silicone hydrogels is 18 per 10,000,^17^, which increases dramatically in extended wear hydrogels to a rate of 29 per 10,000 eyes.^18^ The decision whether to fit hydrogel or silicone hydrogel lenses as extended wear bandage contact lenses is affected by this important factor. Fortunately, re-epithelisation is completed within three to five days after the CXL, and wearing CL is not needed anymore. Corneal re-epithelisation was completed in both groups on the 3^th^ day in this study and CL was removed. None of our cases were found to have microbial keratitis caused by CL or epithelium patency.

Removing the corneal epithelium at the CXL creates an opportunity for microbial agents. Therefore, one should not prefer lens materials that would decrease the speed of epithelisation or prolong it. The related literature encompasses studies that investigated the effects of different lens materials on photorefractive keratectomy (PRK), laser-assisted sub epithelial keratomileusis (LASEK) and other cornea epithelial problems.^19^-^22^ However, studies that compare CL materials after the CXL are quite limited in number.

Considering that keratoconic corneas could frequently be accompanied by allergic eye disease, the CL material to be inserted after the CXL gains more importance. The present study compared two different CLs with different designs and materials. Distribution of preoperative allergic conjunctivitis was close in both CL patient groups, and the difference was not statistically significant. Symptoms and findings indicated no significant differences in both groups after the CXL. However, in cases with allergic conjunctivitis, PE ratio was observed more when CL was removed. This difference in the PE ratio was associated with allergic eye disease rather than the CL material.

### Limitation of the Study

The number of case with allergic conjunctivitis was fewer than other group. It was a limitation for this study. And also, the not evaluated of patients on the 2nd day for corneal re-epithelialization was a limitation.

In conclusion, with the use of Balafilcon A and Hioxifilcon A lens materials, KC patients who underwent the CXL were found to have similar symptoms and clinical findings throughout the re-epithelisation process after the CXL. However, in KC patients accompanied by allergic conjunctivitis, epithelial staining and PE were observed more in the early period after the CXL. It should be kept in mind that epithelial problems and sterile stromal infiltrate are encountered more frequently in the postoperative early period in KC patients who have allergic eye disease findings before the CXL.
